# Determinants of postnatal checkup for newborns in Ethiopia: Further analysis of 2016 Ethiopia demographic and health survey

**DOI:** 10.1186/s12884-020-03468-9

**Published:** 2020-12-07

**Authors:** Abdu Seid, Mohammed Ahmed

**Affiliations:** 1 Department of Midwifery, College of Health Science, Woldia University, Woldia, Ethiopia; 2 Department of Public Health, College of Health Science, Woldia University, Woldia, Ethiopia

**Keywords:** Postnatal checkup, Newborn, Ethiopia

## Abstract

**Background:**

The absence of suitable care during the postpartum period might result in substantial ill-health and even the demise of newborns. So, identifying and intervening thus factors increase postnatal newborn care utilization thereby reducing neonatal mortality. Therefore, this study aimed to ascertain the determinants of the postnatal checkup of a newborn in Ethiopia.

**Method:**

A cross-sectional study was accompanied using the 2016 Ethiopia Demographic and Health Survey (EDHS) data set. The samples were designated by employing a two-stage stratified cluster sampling technique. All statistical analysis were weighted in order to take into consideration complex survey design. Bivariate and multivariate logistic regression analysis was also carried out to examine the association between use of postnatal care of newborn and selected independent variables. Adjusted odds ratios (AOR) were used to state a statistically significant suggestion.

**Result:**

A total of 7091 samples of the reproductive age of newborn mothers were included and analyzed. According to multivariate analysis, the odds of postnatal checkups of the newborn were 2.45 times higher among mothers who had 1–3 ANC visits and 3.42 times higher among mothers who had four and above visits than mother who did not have ANC visit. The odds of postnatal checkups of the newborn were 1.4 times higher among mothers who had access to media compared to their counterparts. Likewise, the odds of postnatal checkups of the newborn were 1.67 times higher among mothers who had delivered in a health facility than who delivered at home.

**Conclusions:**

This study revealed that accessed media, being rich or middle in the wealth index category, having ANC visits, and institutional delivery was positively associated with the utilization of postnatal care checkup of the newborn. Therefore, information education and communication programs should perform a critical role in inspiring mother to take their newborns for postnatal checkup after birth.

## Background

According to WHO and UNICEF recommendations, women who delivered in health institutions and their babies should be evaluated for problems immediately within 24 hours and should provide an appointment to return for further postnatal care, despite all things is going healthy, and provide counseling to coming back instantly if they notice any danger signs [1]. Likewise, if it is home delivery, the first postpartum communication must be as early as possible within 24 hours of birth. Overall, the minimum of three further postpartum checkups is suggested for all mothers and newborns, on day 3 (48–72 hours), between days 7–14 after birth, and 6 weeks after birth [2, 3].

Evidence suggested that the highest proportions of neonatal mortality were found in sub-Saharan Africa and have revealed the sluggish improvement in dropping newborn deaths, particularly deaths in the first week of life. In, African about 1.16 million babies die every year in the first 28 days of life, and 850,000 babies die in the first week of born [4, 5].

Based on the Ethiopian national survey data 2016 report, the neonatal mortality rate was 29% of deaths per 1,000 births [6].

Globally, about 99% coverage of outreach and institution-based medical care interventions of newborn care can prevent about 41–72% of neonatal deaths [7].

Therefore, the absence of suitable care during the postpartum period might result in significant ill-health and even demise of newborns [2]. According to a 2016 EDHS report, only 13% of newborns had received postnatal check in the first two days after birth [6].

Regarding the factors about postnatal checkup of the newborn, different studies showed that antenatal care (ANC) visit during pregnancy, mode of delivery, marital status, wealth index, and distance to the health facility, mother’s education, residence, media access, ethnic group, pregnancy desire, and region were a significant predictor of postnatal care (PNC) of a newborn baby  [8–13]. Moreover, place of delivery, gravidity, parity, and woman’s occupation were determinant factors for PNC services utilization of both the mother and newborn [14, 15].

Generally, the first month of the postpartum period is a life-threatening time in the lives of mother and newborn but, it is the most overlooked time for the provision of qualified health care service for the newborn and mother. Therefore, delivering proper postnatal care is life-threatening to safeguard maternal and newborn health [2, 16–18]. Identifying and intervening thus factors enables postnatal newborn care utilization thereby reducing neonatal mortality. Even though Ethiopia continued to be one the largest sources of maternal and child mortality worldwide, no studies conducted so far to elucidate the factors that perpetuate or hinder postnatal care checkup at the national level. Therefore, this study aimed to identify determinants of postnatal checkup of a newborn in Ethiopia which enables to provide evidence for policy guidelines.

## Methods and materials

### Data

This study was based on a public domain dataset of 2016 Ethiopian demographic and health survey which is a nationwide representative data. There are a total of 84,915 enumerations area (EAs) among them 17,185 are in urban areas and 67,730 in rural areas. The two-stage stratified sampling was employed to collect data. A comprehensive explanations of study design and methods of data collection for the 2016 EDHS is available elsewhere [6]. Data is collected using three main questionnaires which were the men’s questionnaire, the household questionnaire, and the women’s questionnaire. But, our data was taken from the women’s questionnaire which comprises demographic and socioeconomic data on women within their reproductive ages (15–49). All women aged 15–49 years are usual members of the selected households and were eligible for the survey. Afterward rejecting the missing values the total analytic samples of this study were 7091 mothers who have ever given birth in the last five years aged 15–49 years.

### Study variables

The main outcome of interest was the postnatal checkup of newborns. The outcome variable dichotomized as (0 = No and 1 = Yes). The independent variables were carefully chosen from a previous study associated to postnatal checkup of newborn which includes; maternal age, religion, parity, residence, maternal education, media exposure, wealth index, distance to a health facility, mothers occupation, health insurance, antenatal care visit, and place of delivery and size of baby (small, average, and large) [19–25]. Maternal age was recoded as; 15–19, 20–24, 25–34, and 35–49. Likewise, wealth index recoded by merging variables like; (poorest and poorer) as “poor”, middle as “middle” and (richer and richest) also merged as “rich”. Moreover, media exposure were dichotomized based on answer to how often participants read a newspaper, watched television or, listened to the radio. Respondents who answered at least once a week one of the sources were considered to have accessed to media and recoded as yes = 1, and otherwise no = 0. Lastly, mother’s occupation was recoded as not working (0) whereas women having different work types as working (1).

### Statistical analysis

The data analysis was done using all necessary statistical procedures. First, descriptive statistics and weighted percentages of independent variables were computed. Secondly, Bivariate and multivariate logistic regression analysis was also carried out to examine the association between use of postnatal care of newborn and selected independent variables. Adjusted odds ratios (AOR) were used to state a statistically significant suggestion. The analysis was done using SPSS version 24 [26]. The whole statistical analysis were weighted in order to take into consideration complex survey design, using SPSS.

## Result

### Distribution of the characteristics of the study participants

A total of 7091 samples of reproductive age mothers were involved and analyzed. Out of the samples, 6427(90.6%) of mothers did not experience postnatal checkup of their newborn whereas the remaining 664(9.4%) of newborns received postnatal checkup. Among the age category 30–39 years, about 250(35.8%) of respondents were received postnatal checkups. Similarly, 426(71.8%) of rural residents were received postnatal checkups at the time of the survey. Regarding the mother’s educational status, 286(49.8%) of them who had no education were received postnatal checkup. Moreover, 408(55.4%) of the mother who attended 4 and above ANC visits were obtained postnatal checkups.

Besides, bivariate analysis (chi-square test) identified that place of residence, education status of the mother, wealth index, and exposure to mass media, mother occupation, and religion, number of ANC visit, place of delivery, health insurance, and distance to health facility were statistically significantly associated with postnatal care of the newborn baby. **(**Table 1**)**

**Table 1:** Bivariate analysis on determinants of postnatal checkup among the newborn in Ethiopia (*n* = 7091)

Variable	Category	Not received PNC (***n*** = 6427)	Received PNC (***n*** = 664)	Chi-square *p*- value
		No (%)	Yes (%)	
Age (in year)	15–19	325(4.4)	27(4.6)	0.90
	20–29	3136(47.7)	327(4.1)	
	30–39	2385(37.9)	250(35.8)	
	40–49	581(9.9)	60(10.2)	
Residence	Urban	1253(11.3)	238(28.2)	< 0.001
	Rural	5174(88.7)	426(71.8)	
Religion	Orthodox	2004(36.5)	331(4.6)	< 0.001
	Catholic	47(1.0)	2(0.2)	
	Protestant	1217(22.1)	104(18)	
	Muslim	3055(38.1)	220(26.8)	
	Other	104(2.2)	7(0.7)	
Mothers education	No education	4005(64.3)	286(49.8)	< 0.001
	Primary	1697(28.0)	219(31.3)	
	Secondary	479(4.9)	92(12.2)	
	Higher	246(67.0)	67(6.6)	
Occupation	Not working	3742(54.5)	283(45.2)	0.005
	Working	2685(45.5)	381(54.8)	
Wealth index	Poor	3345(45.4)	209(22.9)	< 0.001
	Middle	914(21.0)	97(20.8)	
	Rich	2168(33.6)	358(56.3)	
Media access	No	4286(67.2)	296(46.0)	< 0.001
	Yes	2141(32.8)	371(54.0)	
Number of ANC visit	None	2379(39.2)	66(12.5)	< 0.001
	1–3	1882(30.9)	190(32.1)	
	>= 4	2166(29.9)	408(55.4)	
Place of delivery	Home	497(69.3)	229(40.0)	< 0.001
	Health institution	2330(30.7)	435(60.0)	
Size of newborn baby	Small	1958(32.2)	202(28.3)	0.251
	Average	2675(40.6)	304(45.1)	
	Large	1794(27.2)	158(26.6)	
Distance to the health facility	Big problem	3498(59.7)	240(39.4)	< 0.001
	Not big problem	2929(40.3)	424(60.6)	
Health insurance	No	6216(96.0)	620(93.1)	0.013
	Yes	211(4.0)	44(6.9)	

### Factors associated with a postnatal checkup of the newborn

The whole variables were entered into multivariate logistic regression analysis. After adjusting for potential confounders by logistic regression; media access, wealth index, number of ANC visits, and place of delivery were positively associated with the utilization of postnatal checkups. But, being Catholic, Protestant, and Muslim religious followers were negatively associated with the outcome variable.

In this study, the odds of postnatal checkup of the newborn were 1.4 times (AOR: 1.4; 95% CI: 1.32–1.85) higher among mothers who had access to media than their counterparts. Similarly, the odds of postnatal checkup of the newborn were 1.8 times (AOR: 1.80; 95% CI: 1.24–2.61) higher among mothers who had rich wealth index status and 1.63 times higher among middle wealth index (AOR: 1.63; 95% CI: 1.11–2.39) than mother who had poor wealth index.

Regarding religion, the odds postnatal checkup of the newborn were lower by 82% (AOR: 0.18; 95% CI: 0.42–0.84, 36% (AOR: 0.66; 95% CI: 0.44–0.99), and 32% among Catholic, Protestant and Muslim (AOR: 0.68; 95% CI: 0.49–0.96) religious followers compared than orthodox followers, respectively.

Moreover, the odds of postnatal checkup of the newborn were 2.45 times (AOR: 2.45; 95% CI: 1.59–3.75) higher among mother who had 1–3 ANC visits and 3.42 times (AOR: 3.42; 95% CI: 2.16–5.43) higher among mothers who had four and above visits than mother who did not have ANC visit. The odds of postnatal checkups for the newborn were 1.4 times (AOR: 1.4; 95% CI: 1.32–1.85) higher among mothers who had access to media compared to their counterparts.

Lastly, the odds of postnatal checkups for the newborn were 1.67 times (AOR: 1.67; 95% CI: 1.18–2.36) higher among mothers who had delivered in a health facilities than those delivered at home. **(**Table 2**)**

**Table 2:** Multivariate logistic regression showed determinants of postnatal checkup of the newborn in Ethiopia (*n* = 7091)

Variable	Category	COR(95% CI)	AOR(95% CI)
Age	15–19	1	1
	20–29	0.98(0.55,1.75)	1.092(0.56–2.10)
	30–39	0.90(0.50,1.62)	1.102(0.54–2.24)
	40–49	0.98(0.52,1.83)	1.527(0.74–3.15)
Residence	Urban	3.05(2.23,4.24)	1.06(0.64–1.76)
	Rural	1	1
Religion	Orthodox	1	1
	Catholic	0.11(0.02,0.52)	0.18(0.42–0.84)*
	Protestant	0.55(0.38,0.79)	0.66(0.44–0.99)*
	Muslim	0.47(0.34,0.65)	0.68(0.49–0.96)*
	Other	0.20(0.05,0.89)	0.52(0.12–2.14)
Mothers education	No education	1	1
	Primary	1.44(1.08,1.92)	0.92(0.64–1.31)
	Secondary	3.21(2.25,4.56)	1.09(0.65–1.83)
	Higher	3.16(1.95,5.10)	0.873(0.41–1.87)
Occupation	Not working	1	1
	Working	1.45(1.12,1.88)	1.11(0.84–1.46)
Wealth index	Poor	1	1
	Middle	1.97(1.37,2.81)	1.63(1.11–2.39)*
	Rich	3.33(2.48,4.46)	1.80(1.24–2.61)*
Media access	No	1	1
	Yes	2.39(1.85,3.10)	1.4(1.32–1.85)*
Number of ANC visit	None	1	1
	1–3	3.25(2.19, 4.83)	2.45(1.59–3.75)*
	>= 4	5.81(3.98,8.47)	3.42(2.16–5.43)*
Place of delivery	Home	1	1
	Health institution	3.39(2.62, 4.41)	1.67(1.18–2.36)*
Size of a newborn baby	Small	0.78(0.59,1.04)	0.82(0.6–1.12)
	Average	1	1
	Large	0.88(0.65,1.18)	0.99(0.72–1.35)
Distance to the health facility	Big problem	1	1
	Not big problem	2.27(1.78,2.91)	0.77(0.58–1.02)
Health insurance	No	1	1
	Yes	1.63(0.99, 2.67)	0.96(0.53–1.74)

*show that *p*-value < 0.05

## Discussion

This study looked at the determinants of postnatal checkup of a newborn in Ethiopia based on data from the 2016 EDHS. In this study, the odds of receiving PNC of the newborn within the 28 days of life were greater among women who had rich and middle wealth classes compared to those mothers who had poor wealth index. This result is reinforced by studies done in India [9], Uganda [27], and Rwanda [13]. The consistency might be for the reason that women who have good wealth status might be autonomous to make a decisions on the use of house hold incomes, and they can also afford payments related to health care service to their newborns and themselves [1, 28].

Likewise, this study suggested that access to media by mothers has been found to have a positive association with the use of PNC services among the newborn. This finding is in line with studies done in Uttar Pradesh [29], Uganda [27], and Nigeria [28]. This might be explained by mother’s who have access to media have been found to have increased antenatal care visits, which enables them to know the benefits of PNC after birth [1].

Likewise, this study also suggests that newborns whose mothers who had 1–3, and greater than four ANC visits had higher odds of having postnatal checkup of the newborn than those who did not have antenatal care follow up during pregnancy. This finding is in line with studies done in rural Tanzania [15], Uganda [27], Kenya [8], and the Tigray region in Ethiopia [30]. This could be explained that the provision of counseling and health education to mothers by skilled health care providers at the time of ANC follow up is paramount [31]. Moreover, newborns delivered at the health facility were more likely to receive PNC than those delivered at home which is supported by studies done in rural Uttar Pradesh [29], Nepal [14], Tanzania [15], and Nigeria [32]. This could be justified by mothers delivering at a health facility increases the chances of skilled birth attendance, which plays a vital role in ensuring that mothers and newborns receive comprehensive care, including postnatal care soon after delivery. Another possible explanation for this finding is that delivery at a health facility ensures access to emergency obstetric care to deal with complications during labor, which may also expose women and newborns to postnatal care[27].

Lastly, regarding religion, the odds of postnatal checkups for the newborn among Catholic, Protestant, and Muslim were less likely to utilize newborn care than the orthodox follower. This is finding is supported by a study done in Ethiopia [33] in which being a follower of the orthodox religion associated with postnatal care utilization of mothers and newborns. Even if, we could not find other research to justify, the possible explanation could be due to differences in religious advice among followers to utilize the postnatal care service of the newborn. Nevertheless, it needs further research to explain the differences in PNC utilization of newborns among followers of different religions.

## Conclusions

This study revealed that accessed media, being rich or middle in the wealth index category, having ANC visits, and institutional delivery was positively associated with the utilization of postnatal checkup of the newborn. The researchers recommended that information education and communication programs should perform a critical role in inspiring mothers to take their newborns for postnatal follow up after birth and great emphasis should be given on ANC service to increase the use of postnatal checkup of the newborn.

## Data Availability

For this analysis, researchers were used the USAID–DHS program 2016 Ethiopian demographic and health survey data set. To request the same or different data for another purpose, a research project should be submitted to the DHS program here; https://dhsprogram.com/data/AccessInstructions.cfmThe DHS Program will normally review all data requests within 24–48 hours and send notification if access has been permitted. Afterward receiving consent, the researcher can login and select the specific data in the format they choose.

## Abbreviations

AOR: Adjusted odds ratio
ANC: Antenatal care
COR: Crude odds ratio
CI: Confidence interval
EDHS: Ethiopia Demographic Health Science
PNC: Postnatal care
SPSS: Statistical Package for Social Science
UNICEF: United Nations International Children’s Emergency Fund
WHO: World Health Organization
